# Modulation of the Toll-like Receptor Pathway in Ovine Endometria During Early Pregnancy

**DOI:** 10.3390/ani15070917

**Published:** 2025-03-22

**Authors:** Guoao Yu, Mengyao Song, Chenxu Wu, Xiaoxin Ma, Leying Zhang, Ling Yang

**Affiliations:** School of of Life Sciences and Food Engineering, Hebei University of Engineering, Handan 056038, China; 15933768772@163.com (G.Y.); smy1502073@163.com (M.S.); wuchenxu112@163.com (C.W.); 18632453165@163.com (X.M.); zhangly056000@126.com (L.Z.)

**Keywords:** endometrium, pregnancy, toll-like receptor, sheep

## Abstract

Correct immunological dialogue between the maternal uterus and conceptus is essential during implantation, and Toll-like receptors (TLRs) participate in maternal immune tolerance during pregnancy. This study aimed to analyze the effect of early pregnancy on Toll-like receptor pathways in the ovine endometrium. Ovine endometria were obtained on day 16 of the estrous cycle (N16) and days 13, 16, and 25 of pregnancy (P13, P16, and P25), and expression profiles of TLR members, including TLRs, tumor necrosis factor receptor-associated factor 6 (TRAF6), interleukin 1 receptor-associated kinase 1 (IRAK1), and myeloid differentiation primary response gene 88 (MyD88), were detected by quantitative real-time PCR, Western blot analysis, and immunohistochemistry analysis. The data of this study showed that the expression of TLR2 and TLR5 was gradually increased during early pregnancy compared to N16, and TLR3 expression was greater at P16 and P25 than at N16 and P13. However, the expression levels of TLR4 and TRAF6 were weaker at P13 and P16, and the expression of MyD88 was inhibited by early pregnancy. Furthermore, early pregnancy regulated IRAK1 expression. These findings corroborated that the TLR pathway was modulated in the ovine endometrium during early pregnancy, which may be involved in maternal immunoregulation.

## Simple Summary

Uterine immune regulation is related to pregnancy establishment. Toll-like receptors (TLRs) participate in maternal immune responses during pregnancy. This study aimed to analyze the effect of early pregnancy on TLR members in the ovine endometrium. The results of this study showed that the expression levels of TLR members were modulated, and two TLR member proteins were located in the endometrium in a protein-type-, cell-type-, and pregnancy-status-specific manner, suggesting that the changes in expression of TLR members in the ovine endometrium may be essential for pregnancy establishment during early pregnancy.

## 1. Introduction

In humans, 50% of embryos fail to complete implantation, which is related to an absence of endometrial receptivity that is mediated by uterine immune responses. Therefore, it is essential to elucidate the molecular mechanisms underlying maternal uterine receptivity [1]. An immunological dialogue between the maternal uterus and the conceptus during implantation plays a crucial role in maternal immunological tolerance towards the fetus, but endometrial immune disorder leads to an adverse pregnancy outcome [2]. The fetus carries paternal antigens that do not trigger maternal immune rejection during normal pregnancy, thanks to the sophisticated regulation of maternal–fetal immune activation and immune tolerance [3]. During early pregnancy, many cytokines produced by the endometria are involved in maternal–fetal immunological cross-talk, which is related to embryo attachment and implantation in cattle [4]. In sheep, albumin and claudin 4 and endometrial H3K18 lactylation play key roles in a conceptus–endometrium cross-talk during early pregnancy, which are necessary for cell adhesion and invasion and embryo implantation [5]. Our previous report also revealed that early pregnancy regulated the expression of endometrial nucleotide-binding domain-like receptors, which were related to regulating maternal immune homeostasis and uterus–conceptus interactions in ewes [6]. However, further investigation is needed to explore additional immune signaling pathways involved in regulating uterine immune responses during pregnancy in sheep.

Toll-like receptors (TLRs) are involved in many immune-related processes that are associated with inflammatory, defensive responses, and adaptive immunity via TLR-mediated signal transduction [7]. At the maternal–fetal interface in humans, both immune and non-immune cells, including decidual cells, are present, and their expression patterns change throughout different stages of pregnancy [8]. During early pregnancy, *TLR* mRNA is detected in ovine trophoblasts, and some of the *TLR* mRNA is modulated in the peripheral blood leukocytes, which contributes to the establishment of pregnancy [9]. Placental immune cells express TLRs during normal pregnancy, but disorder of TLR expression has been associated with adverse pregnancy outcomes by upregulating inflammatory cytokines in mice, rats, and humans [10]. In pigs, the endometrium expresses TLRs and myeloid differentiation primary response gene 88 (MyD88) in a stage-specific manner during early pregnancy, which are associated with progesterone and interferon-γ and involved in pregnancy establishment [11]. However, further studies are needed to understand the regulation of uterine immune responses via the TLR signaling pathway during pregnancy in sheep.

Interferon-tau (IFNT) and other conceptus secretory factors play a crucial role in the communication between embryo and endometrium in a paracrine manner, influencing pregnancy establishment in ruminants [12]. There are significant changes in endometrial immune responses, nutrient transport and utilization, and proteasome-dependent proteolysis during implantation through proteomic analysis in ewes [13]. Pregnancy alters the expression of 19 specific miRNAs in the endometrium, which regulate the expression of 315 genes and are related to molecular function and biological processes in sheep [14].

It has been hypothesized that embryonic signals influence TLR expression during implantation, thereby modulating the maternal immune system. For this purpose, the expression profiles of TLR pathway members, including TLR2, TLR3, TLR4, and TLR5, as well as MyD88, tumor necrosis factor receptor-associated factor 6 (TRAF6), and interleukin (IL) 1 receptor-associated kinase 1 (IRAK1), were investigated here at mRNA and protein levels in the ovine endometrium during early pregnancy. The findings of present study will be used to decrease the embryo loss in the bovine and ovine.

## 2. Materials and Methods

### 2.1. Animals and Experimental Design

The exact days of pregnancy or the estrous cycle of the uteruses obtained from slaughter were unknown, and the endometrium includes superficial glandular epithelium, glandular epithelium, and the stroma, etc., so cell lines cannot be used instead. Therefore, small-tail Han ewes were assigned randomly to four groups (*n* = 6 for each group), and estrous behavior was checked using teaser rams. Pregnant animals (three groups) were mated twice 12 h apart using rams with proven fertility, while cyclic animals were mated to vasectomized rams, and the day of estrus was considered as day 0. Ewes were euthanized on days 13 (P13), 16 (P16), and 25 (P25) of pregnancy, as well as on day 16 of the estrous cycle (nonpregnancy, N16). Pregnancy was verified by the appearance of embryonic trophoblasts in the uterus. Endometrial tissue pieces (0.5 cm^3^) from the uterine body (including uterine caruncle) were collected and fixed with fresh 4% paraformaldehyde and also rapidly frozen at −80 °C until RNA isolation and protein analysis.

### 2.2. RNA Extraction and RT-qPCR Assay

The endometrial samples were homogenized in 1 mL TRNzol Universal Reagent (Tiangen Biotech Co., Ltd., Beijing, China) according to the manufacturer’s protocol to isolate the total RNA. Quantity and quality of the total RNA were determined using a Nanodrop (ND-100, Thermo Fisher Scientific, Waltham, MA, USA), and oligonucleotide primers (as described previously [15]) for the target genes and the reference gene (glyceraldehyde-3-phosphate dehydrogenase, *GAPDH*) were designed and synthesized by Shanghai Sangon Biotech Co., Ltd. (Shanghai, China). A FastQuant RT kit (with gDNase, KR106, Tiangen Biotech Co., Ltd., Beijing, China) was used to remove genomic DNA and synthesize first-strand complementary DNA. Quantitative PCR reactions were set up as described previously [15]. Reaction specificity and amplification products were evaluated after separation on a 2% agarose gel. The qPCR efficiency for specific gene product amplifications was between 95 and 105%. For fold-change calculation, the mean threshold cycle (Ct) values obtained from the mean of the N16 were used as reference points, and the Ct values of all groups were utilized to analyze fold changes from reference points using the 2^−ΔΔCt^ method [16].

### 2.3. Western Blot Analysis

Endometrial lysate was prepared by frozen ovine endometrial tissue, which was mixed with RIPA buffer (BL504A, Biosharp, Hefei, China), including phosphatase and protease inhibitors. Protein concentrations were measured utilizing a BCA Protein Assay Kit (Tiangen Biotech Co., Ltd., Beijing, China), and 10 μg/lane of total proteins was resolved on polyacrylamide gel by electrophoresis under reducing conditions, followed by transfer to a polyvinylidene difluoride membrane (Millipore, Bedford, MA, USA) in the presence of 20% methanol. The membranes were blocked using 5% skim milk powder for one hour at room temperature to reduce nonspecific binding and then incubated with primary antibodies (Table 1) overnight at 4 °C. The primary antibodies were validated and considered to be cross-reactive species by specific binding to native ovine proteins as described previously [15]. After being washed, the membranes were added to the secondary antibodies (horseradish peroxidase-conjugated (HRP) anti-mouse IgG (BL001A, Biosharp, Hefei, China) or anti-rabbit IgG (BL003, Biosharp, Hefei, China)) and diluted at 1:2000 for one hour at room temperature. A pro-light HRP chemiluminescence kit (Tiangen Biotech Co., Ltd., Beijing, China) was used to visualize the protein bands. Quantity One V452 (Bio-Rad Laboratories, Hercules, CA, USA) was employed to calculate the band intensities, and an anti-GAPDH antibody (Table 1) was utilized in the same conditions as described for primary antibodies to normalize expression values.

### 2.4. Immunohistochemistry Analysis

Isolated uterine tissues were embedded in paraffin and cut into 5 μm thick sections. Uterine sections stained with hematoxylin and eosin (HE) were imaged using a light microscope (Nikon Eclipse E800, Tokyo, Japan). Histological sections of the uterus on silanized charged slides were treated with boiling citrate for antigen retrieval, and the sections underwent quenched endogenous peroxidase activity with 3% H_2_O_2_. Nonspecific binding was blocked with 5% normal goat serum in PBS and then subjected to immunohistochemical analysis using the anti-TLR4 antibody (Santa Cruz Biotechnology, Santa Cruz, CA, sc-293072, 1 µg/mL) and anti-MyD88 antibody (Santa Cruz Biotechnology, Santa Cruz, CA, sc-136970, 1 µg/mL) overnight at 4 °C. After being washed three times, incubation with the secondary antibody (HRP conjugated goat anti-mouse (BL001A, Biosharp, Hefei, China)) diluted at 1:500 was performed for one hour at room temperature, followed by the application of a DAB kit (Tiangen Biotech Co., Ltd., Beijing, China) as the chromogen. The sections were counterstained using hematoxylin. The negative control was prepared by substituting the primary antibody with an antiserum-specific isotype at the same protein concentration. Images were acquired utilizing a light microscope (Nikon Eclipse E800, Tokyo, Japan), and quantitative analysis of the immunohistochemical expression of TLR4 and MyD88 proteins in the uterus was conducted by assigning an immunoreactive intensity of a scale of 0 to 3, as described previously [15].

### 2.5. Statistical Analysis

Each group consisted of six replicates, and the data were presented as the mean ± SEM. Statistical analysis of the data was carried out by least-squares ANOVA using Mixed and General Linear Model procedures of the Statistical Analysis System (SAS Institute, Cary, NC, USA). Results were analyzed for the main effects of day and status (cyclic or pregnant) and interaction between day and status, and multiple comparison was performed using the Duncan method. The results were deemed significant at *p* < 0.05.

## 3. Results

### 3.1. Expression of TLR2, TLR3, TLR4, TLR5, IRAK1, TRAF6, and MYD88 mRNAs in the Endometrium During Pregnancy

**Figure 1:**
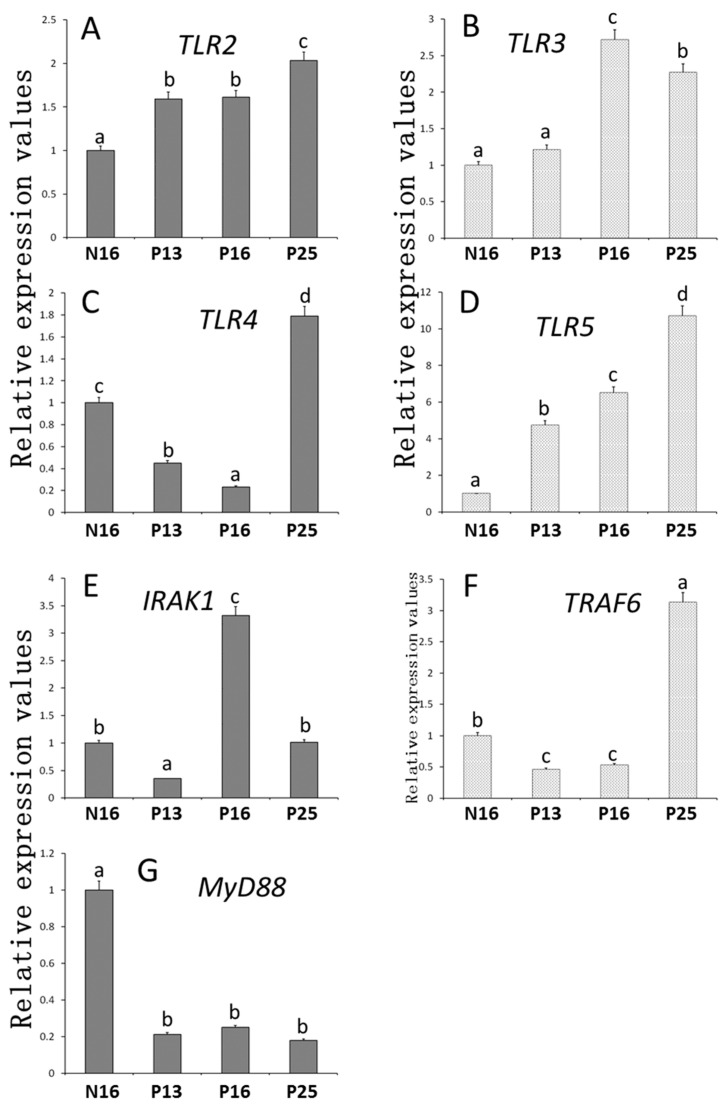
Relative expression values of (**A**) *TLR2*, (**B**) *TLR3*, (**C**) *TLR4*, (**D**) *TLR5*, (**E**) *IRAK1*, (**F**) *TRAF6*, and (**G**) *MYD88* mRNA in the endometrium. Significant differences (*p* < 0.05) are indicated by different letters (a, b, c, and d).

The relative abundances of TLR members were analyzed in Figure 1. The expression patterns of *TLR2* and *TLR5* mRNA (Figure 1A,D) were almost similar, and the abundances of *TLR2* and *TLR5* mRNAs increased from P13 to P25 compared to N16 (*p* < 0.05). On the other hand, the abundance of *TLR3* (Figure 1B) increased on P16 and P25 compared to N16 and P13 (*p* < 0.05), with a peak at P16. Furthermore, the abundances of *TLR4* and *TRAF6* mRNAs (Figure 1C,F) decreased at P13 and P16 compared to N16 (*p* < 0.05) but increased at P25 compared to the other three groups (*p* < 0.05). Nevertheless, the abundance of *IRAK1* (Figure 1E) was the lowest on P13 but was the highest at P16 compared to N16 and P25 (*p* < 0.05). On the other hand, the abundance of *MyD88* mRNA (Figure 1G) was downregulated during early gestation compared to N16 (*p* < 0.05).

### 3.2. Expression of TLR2, TLR3, TLR4, TLR5, IRAK1, TRAF6, and MyD88 Proteins in the Endometrium During Pregnancy

**Figure 2:**
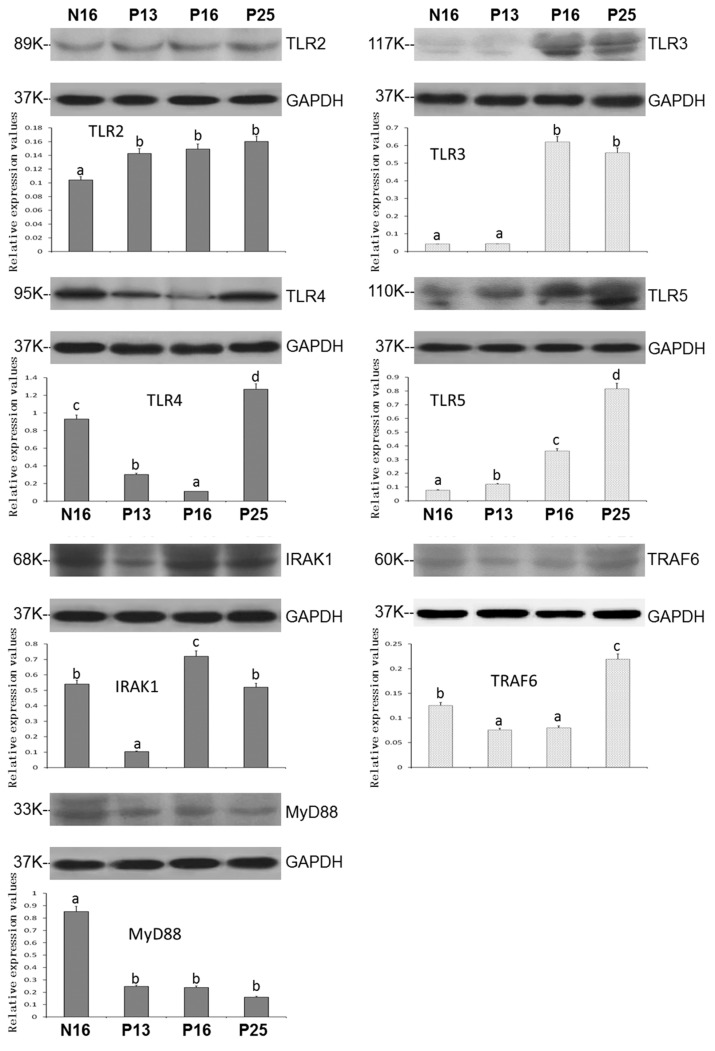
Expression of TLR2, TLR3, TLR4, TLR5, IRAK1, TRAF6, and MyD88 proteins in the endometrium. Significant differences (*p* < 0.05) are indicated by different letters (a, b, c, and d).

Figure 2 and Figure S1 showed that the expression patterns of TLR pathway proteins were similar to the patterns of mRNAs. TLR3 and TLR5 showed some double bands, and the double bands were quantified together. The values of TLR2 and TLR5 were upregulated at P13, P16, and P25 compared to N16 (*p* < 0.05; Figure 2) and reached a peak at P25 for the TLR5 protein. The TLR3 protein levels were higher at P16 and P25 than at N16 and P13 (*p* < 0.05), and there was only a slight expression of the TLR3 protein at N16 and P13. Furthermore, the protein levels of TLR4 and TRAF6 were lower at P13 and P16 than at N16 and P25 (*p* < 0.05) and peaked at P25. Nevertheless, the IRAK1 protein level was downregulated at P13 but upregulated at P16 compared to N16 and P25. In addition, early gestation inhibited the expression of the MyD88 protein in the endometrium (*p* < 0.05; Figure 2).

### 3.3. Immunohistochemistry for TLR4 and MyD88 Proteins in the Endometria

**Figure 3:**
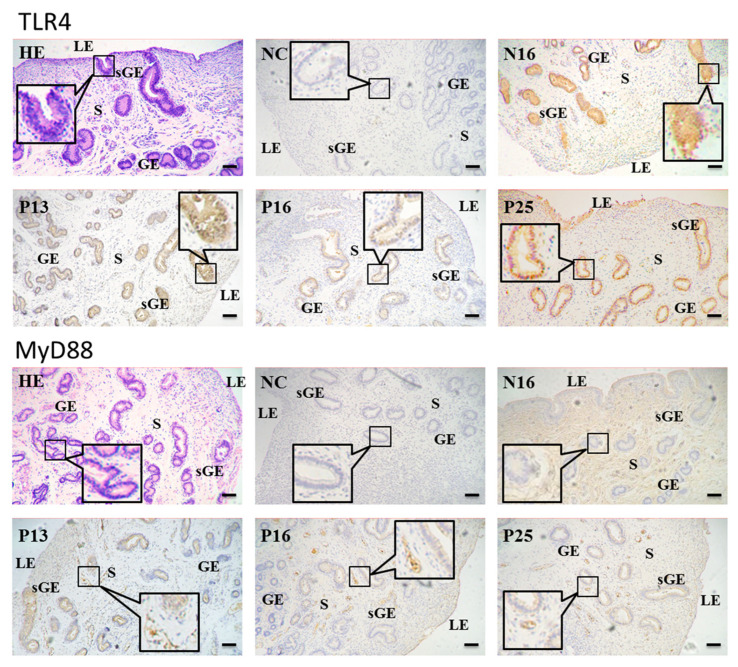
Representative immunohistochemical localization of TLR4 and MyD88 proteins in the ovine endometrium. The TLR4 protein was mainly located in the superficial glandular epithelium (sGE) and glandular epithelium (GE), and the MyD88 protein was mainly limited to the stroma (S), with weak signals in sGE and GE. HE = stained by hematoxylin and eosin; LE = luminal epithelium; NC = negative control; N16 = day 16 of the estrous cycle; P13 = day 13 of pregnancy; P16 = day 16 of pregnancy; P25 = day 25 of pregnancy. Bar = 20 µm.

The immunohistochemistry showed that the TLR4 protein was abundant in the uterine superficial glandular epithelium (sGE) and glandular epithelium (GE). On the other hand, the MyD88 protein was mainly located in the stroma (S), with weak signals in sGE and GE (Figure 3). For the negative control, N16, P13, P16, and P25, the staining intensities for the TLR4 protein were 0, 2, 1, 1, and 3 and for the MyD88 protein were 0, 2, 1, 1, and 1 (Figure 3).

## 4. Discussion

**Figure 4:**
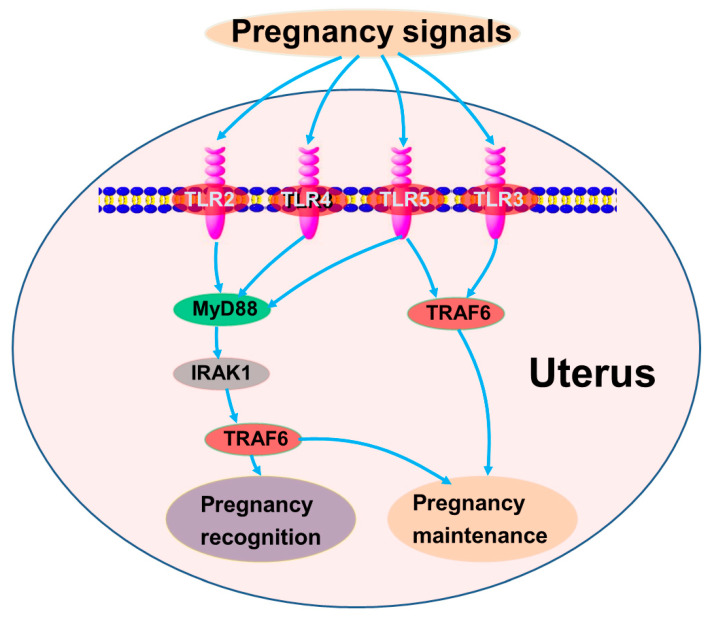
Sketch of TLR signaling pathways in maternal endometrium during early pregnancy. Green, stimulators; pink, negative regulators; gray, changed.

Data from this study indicated that early gestation modulated the TLR pathway. Kawai and Akira (2010) reported that TLR2, TLR4, and TLR5 are implicated in immune responses via MyD88, IRAK1, and TRAF6, and TLR3 and TLR4 participate in immune regulation via TRAF6 in mice and humans [17]. In this study, the expression of TLR2, TLR3, and TLR5 was great during pregnancy, which may downregulate the expression of MyD88. In addition, there was a downregulation of TLR4 at P13 and P16, which may lead to a decrease in the expression of TRAF6 in the uterus. Therefore, it is suggested that early gestation may regulate the expression of TLR pathway members via MyD88-dependent and MyD88-independent (TRAF6) pathways, which may be essential for the recognition and establishment of pregnancy (Figure 4).

The function of TLR2 is mainly related to innate immune cells, but TLR2 signaling also has direct effects on disease progression in endothelial cells [18]. Our previous study showed that *TLR2* mRNA and protein levels were increased in the ovine lymph nodes during early pregnancy, suggesting that TLR2 was related to immunoregulation during pregnancy [19]. Higher *TLR2* mRNA expression is observed in endometrial tissues during the perimenstrual period, whereas lower levels are detected during the periovulatory period, primarily in epithelial and stromal cells in humans [20]. Furthermore, estradiol suppresses TLR2 expression in the vaginal epithelial cell line [21], and it is known that the serum estradiol levels are higher at day 16 of nonpregnancy in ewes. Moreover, *TLR2* is found upregulated in the porcine endometrium during pregnancy, which is related to conceptus interferon-γ that has a critical effect on the pregnancy establishment of pigs [11]. On the other hand, the *TLR2* level is increased during early and late stages of luteolysis and during early gestation in the corpus luteum, which is related to the anti-luteolytic mechanism in ewes [22]. Our data showed that the expression of TLR2 was upregulated in the ovine endometrium, which may be associated with low levels of estradiol, and contributes to pregnancy establishment.

TLR3 signaling can limit chlamydial spread and maintain the integrity of epithelial barrier function during genital tract infection in mice [23]. In addition, *TLR3* mRNA is upregulated in the uterus and cervix in pregnant mice, serving as a protective mechanism for responding to pathogens [24]. Furthermore, the expression level of TLR3 is higher in decidua from normal pregnancies compared to preeclampsia, but there is a contrary result in placental trophoblasts, suggesting that TLR3 is involved in local inflammatory regulation at the maternal–fetal interface in humans [25]. Moreover, it has been reported that the expression value of TLR3 increases in early gestation in the ovine spleen, suggesting that TLR3 is related to maternal immune tolerance [26]. On the other hand, *TLR3* mRNA expression is downregulated in patients with endometrial disease compared to the normal endometrium, and TLR3 protein is mainly located in glandular and luminal epithelium in humans [27]. This study revealed that expression of TLR3 increased at P16 and P25 in the ovine endometrium, suggesting that this change may have a protective effect on the endometrium during pregnancy.

TLR4 can regulate the production of pro-inflammatory cytokines via MyD88-dependent and MyD88-independent pathways, playing a key role in normal pregnancy, but disorder of TLR4 results in adverse outcomes in mice and humans [28]. In addition, malaria infection results in TLR4 activation during pregnancy, leading to a local inflammatory response in the placenta and adverse pregnancy outcomes in mice [29]. Furthermore, the activation of TLR4 promotes the senescence of placental mesenchymal stem cells, which is associated with damage in placental angiogenesis, and contributes to preeclampsia-like manifestations in rats [30]. Moreover, a high-cholesterol diet leads to excessive hypercholesterolemia during pregnancy in rats, which enhances TLR4 activity in uterine arteries, resulting in uterine artery dysfunction and perinatal complications [31]. However, fetal TLR4 plays a protective role in trophoblast cell migration and counteracting placental insufficiency during malaria infection via the Toll/IL-1R-domain-containing adaptor-inducing interferon-β pathway [32]. This research demonstrated that *TLR4* mRNA and protein downregulated at P13 and P16 but upregulated at P25. Thus, it is suggested that the decrease in P13 and P16 may help to initiate implantation, but the increase in P25 may contribute to pregnancy maintenance.

TLR5 is expressed in a lot of types of cells, playing a role in inflammatory cytokine production and being involved in both innate and adaptive immunity [17]. In addition, transcription of *TLR5* in the endometrium of pregnant cows significantly increases from the first to the second and third trimesters [33]. Furthermore, *TLR5* expression levels in the uterus rise with increasing doses of progesterone in pigs [11]. Moreover, high serum progesterone levels are observed during early gestation, and the pregnancy-associated glycoprotein level is upregulated from day 20 of ovine gestation [34]. This study revealed that TLR5 expression levels were gradually increasing from P13 to P16 and P25. On the other hand, our previous study also indicated that TLR5 expressed in the ovine spleen upregulates during early pregnancy [26], which is largely consistent with the present study. Thus, the increasing expression of TLR5 during early pregnancy may be associated with the high level of progesterone in the plasma of ewes.

IRAK1 is an important mediator in IL-1/TLR signaling pathways, inducing inflammatory target gene expression through its kinase and adaptor function [35]. In addition, p-IRAK1 protein expression is upregulated in fetal membranes from females delivering preterm compared to no-chorioamnionitis controls, suggesting that IRAKI is involved in inflammation-induced preterm birth [36]. Furthermore, it has been reported that miR-146b-5p suppresses trophoblast proliferation and implantation-associated inflammation via inhibiting IRAK1 expression, which results in miscarriage in humans [37]. Moreover, conceptus trophectoderm cells express *IRAK1* mRNA, which is implicated in the production of IFNT from the trophectoderm, and IFNT is related to ovine pregnancy recognition on P16 [38]. On the other hand, our previous study also reported that that the IRAK1 protein peaked in the maternal liver at P16 and then returned to the basal level at P25, suggesting that the peak of IRAK1 expression at P16 was related to pregnancy recognition in ewes [15]. Thus, the peak of IRAK1 expression at P16 may be associated with pregnancy recognition, and returning to the basal level at P25 may be helpful for pregnancy establishment.

TRAF6 controls multiple biological processes, including development, organogenesis, immunity, and inflammation [39]. In addition, M1 macrophages can suppress trophoblast invasion and migration, leading to recurrent spontaneous abortion, which occurs indirectly by inhibiting *TRAF6* expression at the mRNA level in mice [40]. Furthermore, TRAF6 participates in modulating zeste homolog 2 ubiquitination and leads to the inhibition of trophoblast cell glycolysis and disorder of M2 macrophage polarization, resulting in recurrent spontaneous abortion in humans [41]. Moreover, umbilical cord mesenchymal stem cell exosomes can modulate intercellular signaling and alleviate placental dysfunction by suppressing TRAF6 expression and NF-κB signaling, thereby rescuing inflammation and inhibiting apoptosis in the mouse placenta [42]. On the other hand, serum exosomal miR-410-3p from spontaneous abortion patients can suppress trophoblast cell migration and invasion as well as the p38 mitogen-activated protein kinase signaling pathway through suppressing *TRAF6* mRNA expression in mice, which contributes to aggravated embryo absorption [43]. Previous studies also reported that the TRAF6 protein increases during early pregnancy in the maternal liver, spleen, and lymph nodes, suggesting that the increase in TRAF6 expression favors maternal immunological tolerance and pregnancy maintenance in ewes [15,19,26]. Our results showed that TRAF6 expression was increased during pregnancy, and this was consistent with the data in the liver, spleen, and lymph nodes [15,19,26], suggesting that the upregulation of TRAF6 may be beneficial for implantation and pregnancy maintenance.

MyD88 plays a crucial role in the secretion of inflammatory cytokines, and TLR/MyD88 signaling is implicated in modulating innate and adaptive immunity [44]. In addition, it is through downregulating TLR4/MyD88/NF-κB signaling that myrtenol reduces lipid peroxidation and inflammatory responses in rats [45]. Furthermore, sinomenine can lessen inflammation by suppressing TLR4/MyD88/NF-κB signaling during pregnancy in female rats [46]. Moreover, fetal growth restriction (FGR) upregulates the level of MyD88 protein in maternal rats’ serum, and the suppression of MyD88-related pathways can alleviate FGR [47]. Lipopolysaccharide exposure leads to the upregulation of *MyD88* mRNA at the early blastocyst implantation site, and the inhibition of MyD88-related signaling can be used to prevent infection-induced pregnancy defects in mice [48]. Previous reports also demonstrated that the MyD88 protein was downregulated in the maternal thymus during early gestation, which was associated with the downregulation of cytokines and chemokines in sheep [49]. This study revealed that there was a decrease in the MyD88 level in the endometria, which may lead to a decrease in levels of cytokines and chemokines during early gestation in ewes.

## 5. Conclusions

The data of this study revealed the pattern of the TLR pathway in ovine endometria during the peri-implantation period of pregnancy. Early gestation enhanced the expression of TLR2, TLR3, TLR4, and TLR5 but suppressed the expression of MyD88. Furthermore, IRAK1 expression was modulated, and the expression of TRAF6 was found to be increased. These data provide strong evidence that members of the TLR pathway may be modulated by early pregnancy via MyD88-dependent and MyD88-independent (TRAF6) pathways, which may be critical to the establishment of pregnancy in sheep. The findings of present study may be used to investigate endometrial receptivity and decrease the embryo loss during pregnancy in ruminants. Further studies may explore the expression of TLR in endometrium during mid- and late pregnancy in cattle and sheep.

## Figures and Tables

**Table 1:** Antibodies used for Western blot.

Antibody	Number	Source	Concentrations
Rabbit anti-TLR2 polyclonal antibody	ab191458	Abcam, Cambridge, UK	0.2 µg/mL
Mouse anti-TLR3 monoclonal antibody	sc-32232	Santa Cruz Biotechnology, Santa Cruz, CA, USA	0.2 µg/mL
Mouse anti-TLR4 monoclonal antibody	sc-293072	Santa Cruz Biotechnology, Santa Cruz, CA, USA	0.2 µg/mL
Mouse anti-TLR5 monoclonal antibody	sc-517439	Santa Cruz Biotechnology, Santa Cruz, CA, USA	0.2 µg/mL
Mouse anti-MyD88 monoclonal antibody	sc-136970	Santa Cruz Biotechnology, Santa Cruz, CA, USA	0.2 µg/mL
Mouse anti-TRAF6 monoclonal antibody	sc-8409	Santa Cruz Biotechnology, Santa Cruz, CA, USA	0.2 µg/mL
Rabbit anti-IRAK1 polyclonal antibody	ab137327	Abcam, Cambridge, UK	0.2 µg/mL
Mouse anti-GAPDH monoclonal antibody	sc-47724	Santa Cruz Biotechnology, Santa Cruz, CA, USA	0.2 µg/mL

## Data Availability

Data supporting the findings of this study are available within the paper.

## Supplementary Materials

The following supporting information can be downloaded at https://www.mdpi.com/article/10.3390/ani15070917/s1, Figure S1: Original Western blot for Figure 2.
