# Supplementary figures and images for: Modulation of the Toll-like Receptor Pathway in Ovine Endometria During Early Pregnancy
Source: Animals (Basel). 2025 Mar 22;15(7):917. doi: 10.3390/ani15070917 (PMC11987747; doi:10.3390/ani15070917)

Figure S1    Original Western Blot

WB full membrane for Figure 2

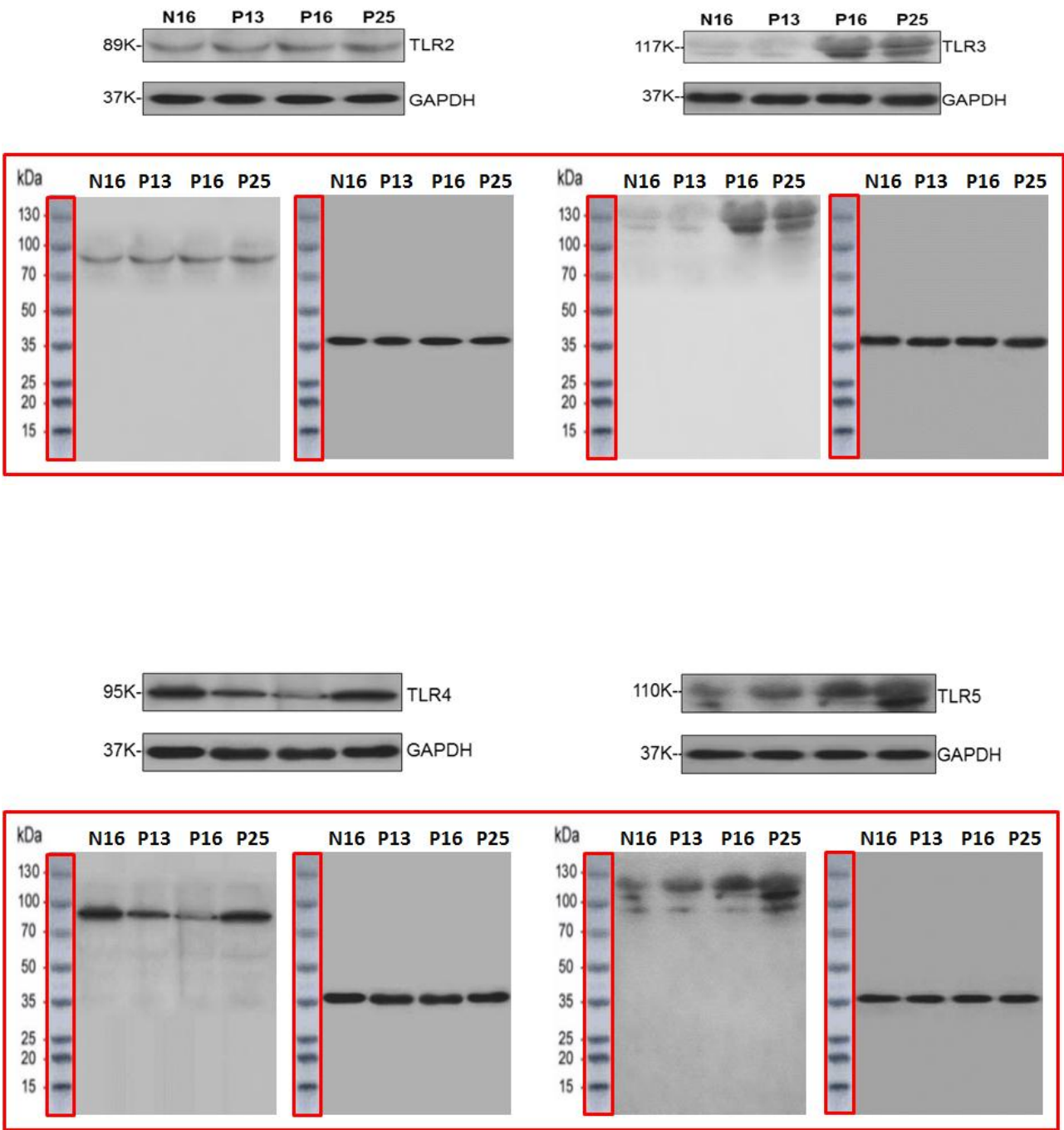

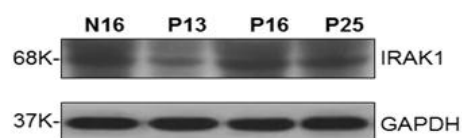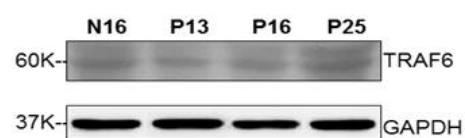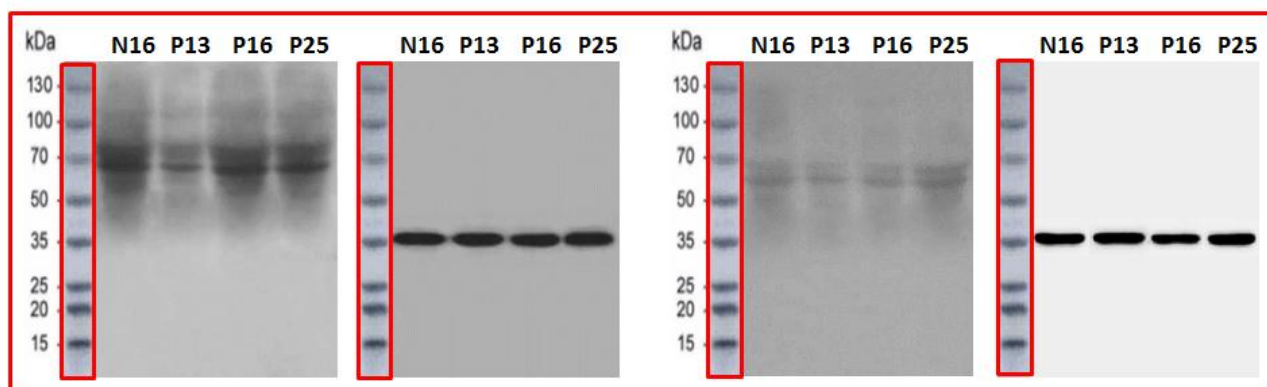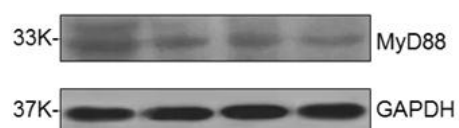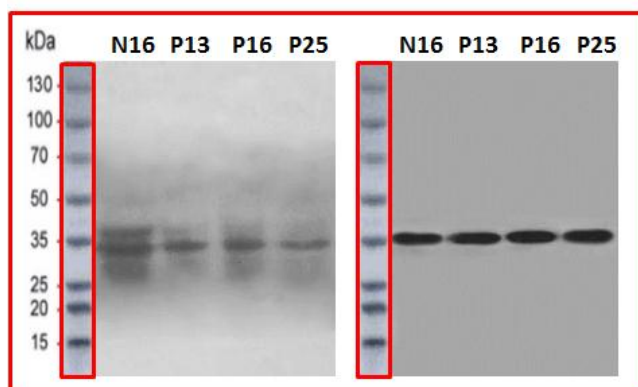

Supplement: Supplementary file 1 [file animals-15-00917-s001.zip › animals-3386554-supplementary.pdf]
